# Imaging, Endoscopic and Genetic Assessment of Marfan Syndrome Presenting with Sigmoid Volvulus: A Review

**DOI:** 10.7759/cureus.619

**Published:** 2016-05-21

**Authors:** Faisal Inayat, Abu Hurairah, Faiq Shaikh

**Affiliations:** 1 Department of Medicine, New York-Presbyterian Hospital, Weill Cornell Medical College, New York City, NY, USA; 2 Division of Gastroenterology, Department of Medicine, SUNY Downstate Medical Center, Brooklyn, NY, USA; 3 Imaging Informatics, University of Pittsburgh Medical Center, Pittsburgh, PA.; 4 Molecular Imaging, Cellsight Technologies, Inc., San Francisco, CA.

**Keywords:** sigmoid, volvulus, marfan, imaging, endoscopic, genetic

## Abstract

The Marfan syndrome (MFS) is a pleiotropic, autosomal dominant disorder of connective tissue with highly variable clinical manifestations. It primarily involves the skeletal, cardiovascular, and ocular systems; however, gastrointestinal complications are rare. Herein, we describe the case of a 31-year-old male who initially presented with acute abdominal pain for one day. His imaging features revealed a dilated sigmoid colon, consistent with sigmoid volvulus that was immediately decompressed. Surgical resection was recommended to treat the sigmoid volvulus. Preceding the treatment, the patient underwent an extensive workup, including an echocardiography that revealed aortic root dilatation. His clinical history, physical exam, and echocardiographic findings raised the suspicion for MFS. Subsequently, the diagnosis of MFS was confirmed on genetic testing. This is a case that highlights the multidisciplinary (clinical, radiological, endoscopic, molecular/genetic) approach to diagnose a patient with MFS who presented with symptomatic sigmoid volvulus. As this presentation may be a harbinger of more severe manifestations of MFS, it is important to identify it as such in order to accomodate for timely management.

## Introduction

Marfan syndrome (MFS) is a connective tissue disorder caused by the mutation of fibrillin-1 gene. While this syndrome is not uncommon, it is rare for it to present with gastrointestinal involvement. Sigmoid volvulus (SV) is an emergent gastrointestinal condition associated with high mortality. The risk factors for SV include chronic constipation, colonic dysmotility, old age, colon cancer, and Hirschsprung’s disease. However, SV in association with MFS is a rare clinicopathologic entity. A review of the literature suggests that only one such case has been reported [1].

## Case presentation

A 31-year-old male presented to the emergency department of our medical center with a one-day history of acute abdominal pain, which was constant, diffuse, non-radiating, stabbing in nature, and aggravated with movement. His last regular bowel motion was one week ago and he had been passing only mucus since. His past medical history was noncontributory and he had no pre-existing gastrointestinal disturbances.

**Figure 1:**
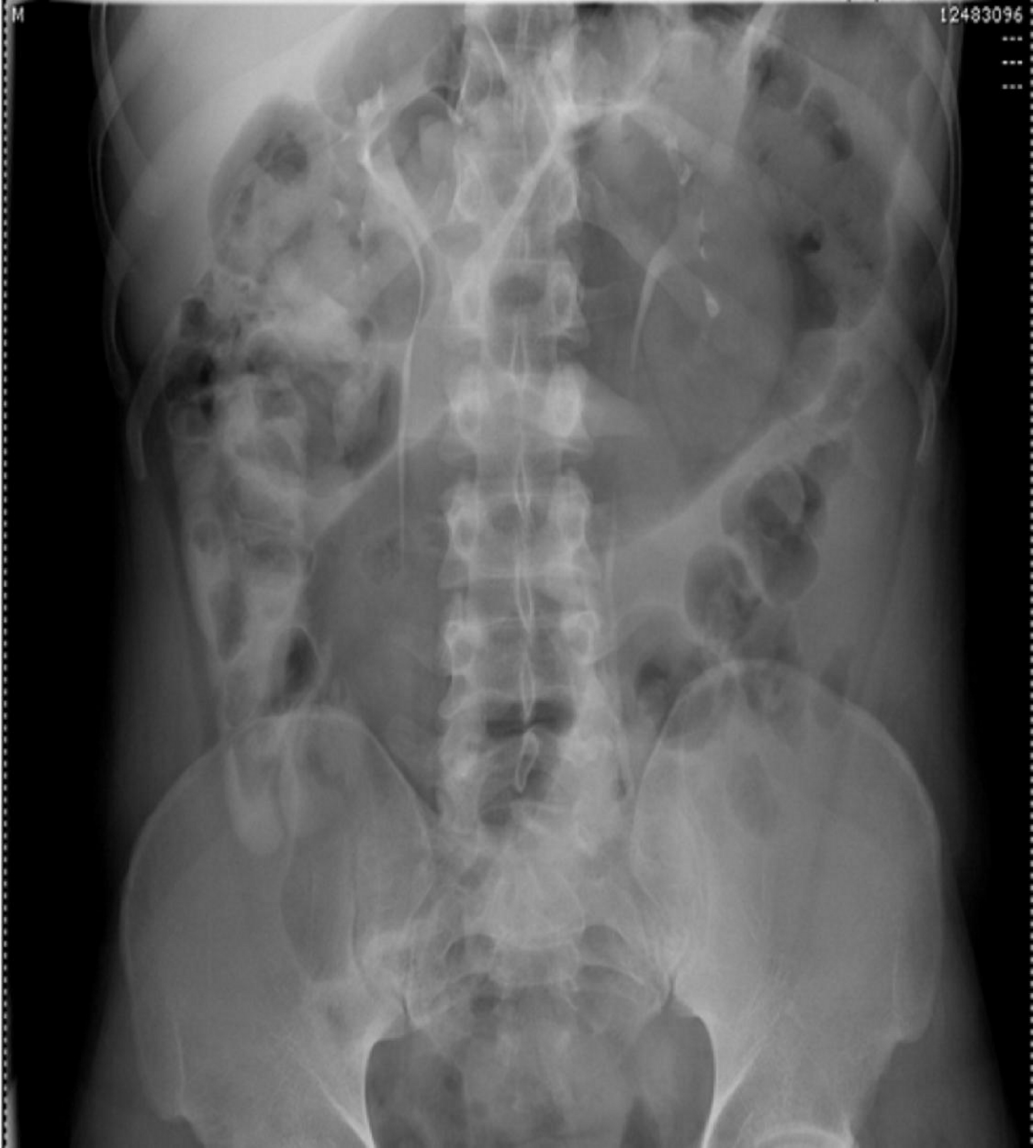
Plain Film Radiograph Anteroposterior view abdominal X-ray demonstrating marked dilatation of the sigmoid colon.

**Figure 2:**
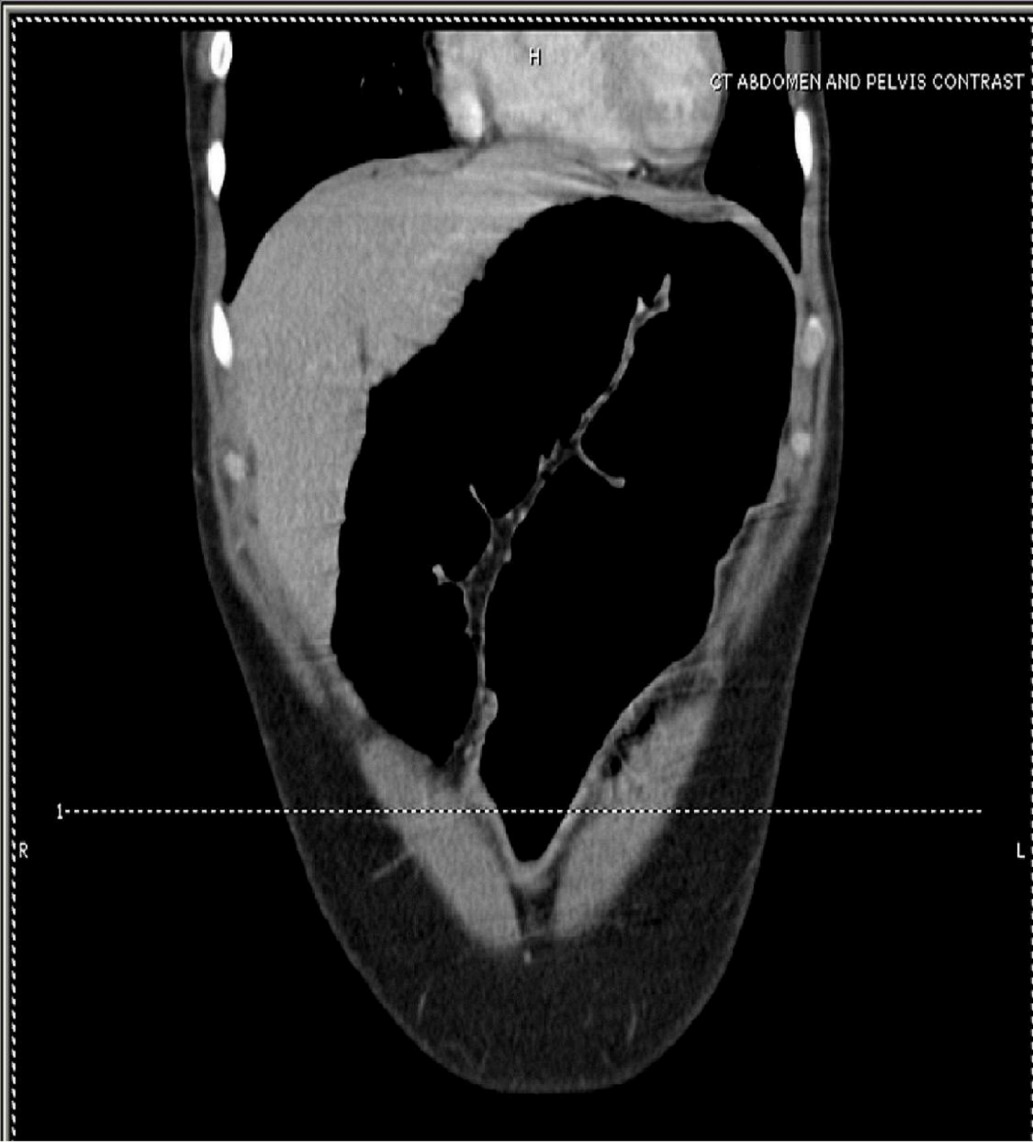
CT of the Abdomen Coronal image of the contrast-enhanced CT of the abdomen showing a significantly distended sigmoid colon looped in an inverted "U" without haustral markings.

Physical examination revealed hypoactive bowel sounds and diffuse abdominal tenderness with rebound. There was no abdominal rigidity or guarding. Rectal examination revealed an empty rectal ampulla. Plain abdominal radiograph demonstrated a greatly dilated sigmoid colon that almost filled the entire abdomen (see Figure 1). A computed tomography (CT) scan of the abdomen revealed a markedly distended ahaustral sigmoid colon looped in an inverted U; the limbs of the sigmoid loop converged toward the pelvis, while the other end entered the left upper quadrant (see Figure 2).

These features were consistent with sigmoid volvulus. Immediate bedside proctoscopy reduced the volvulus and a soft rectal tube was placed thereafter.

**Figure 3:**
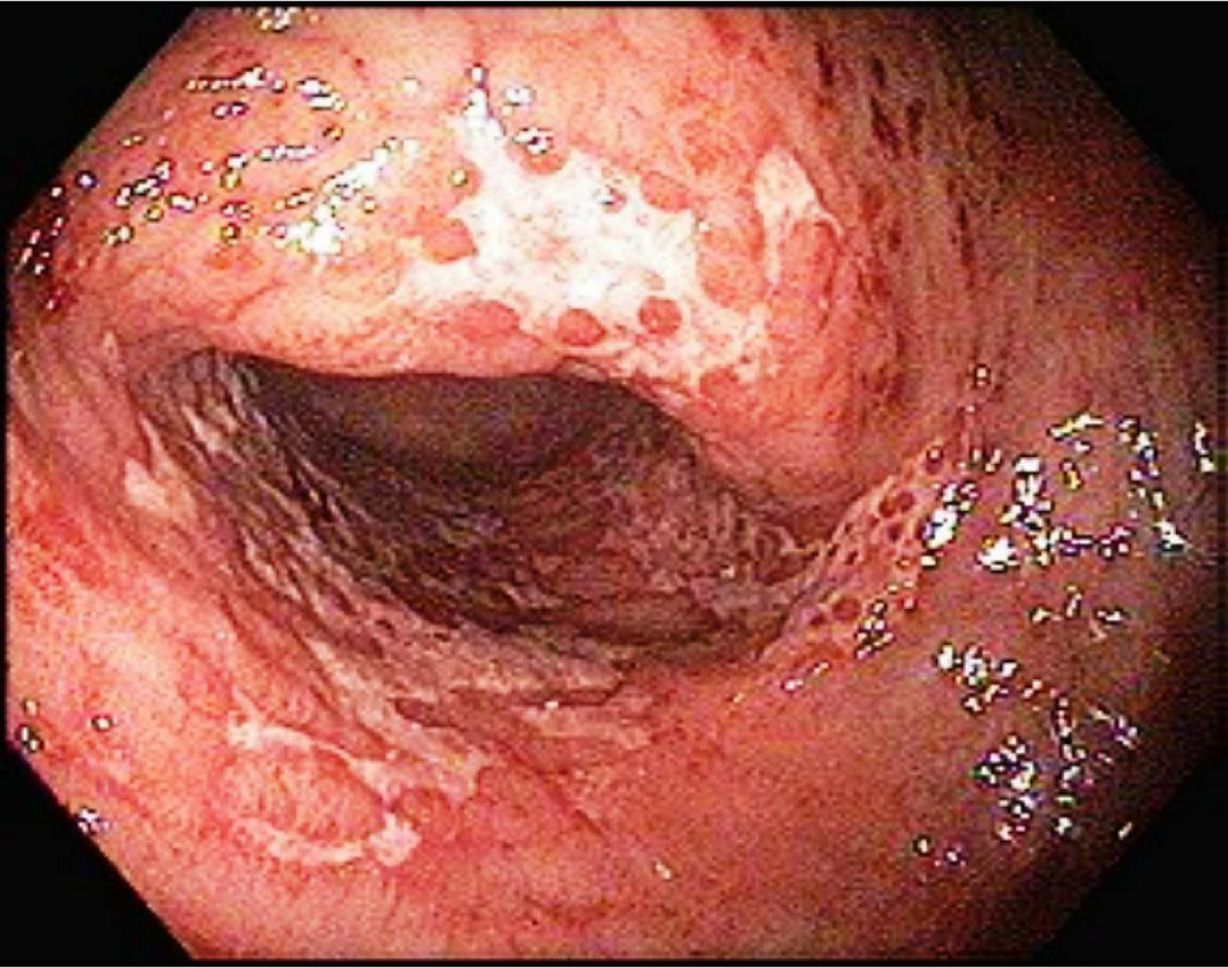
Sigmoidoscopy Sigmoidoscopy showing erythematous mucosal change with ulcerations 30 cm from the anal verge.

Subsequently, flexible sigmoidoscopy was performed to assess for an anatomic etiology and it revealed an erythematous mucosal change with ulcerations 30 centimeters (cm) from the anal verge (see Figure 3). Specimens were collected for biopsy and malignancy was excluded on histopathologic evaluation of the biopsied tissue specimen. Definitive surgery was recommended to avoid recurrent episode of sigmoid volvulus.

During the preoperative workup, the patient was found to be 189 cm tall with an arm span of 204 cm. He had an arm span/height ratio of 1.08. His face appeared narrow and thin, his teeth overcrowded, and he was found to have myopic vision on the visual acuity test. Although Marfan syndrome had not been clearly documented in the patient’s family, it was revealed that his father died at a young age due to a sudden cardiac arrest. Furthermore, he reported of Marfanoid appearance with arachnodactyly in one of his brothers. Laboratory evaluation was notable only for leukocyte count of 10.3 x 10^9^/L. The patient had no signs and symptoms of sepsis and his lactic acid level was within normal limits. Echocardiography showed evidence of aortic root dilatation with mild aortic regurgitation.

Subsequently, a molecular genetic testing was performed and that showed a c.1948 C>T (p.Arg650Cys) mutation in fibrillin-1 gene, confirming the diagnosis of MFS. Genetic screening within the family revealed that his brother was also a carrier of the same mutation. Subsequently, our patient underwent an uneventful subtotal colectomy. His recovery was unremarkable and he was discharged from the hospital. Informed consent was obtained from the patient for this study.

## Discussion

Sigmoid volvulus (SV) occurs when there is torsion of the sigmoid colon over its vascular pedicle, causing obstruction or ischemia [1-2]. Risk factors for sigmoid volvulus include an elongated and redundant sigmoid colon, chronic constipation, obstipation, colonic dysmotility, old age, pregnancy, colon cancer, Parkinson’s, and Hirschsprung’s disease [3]. However, it is extremely rare to find SV secondary to MFS.

MFS is a multisystem connective tissue disorder. It follows an autosomal dominant pattern of inheritance with around 30% of cases showing sporadic mutations [4]. It is caused by mutation of the gene encoding glycoprotein fibrillin-1 (FBN-1), which is the main constituent of microfibrils in the extracellular matrix. Abnormal FBN-1 protein leads to laxity and weakness of the tissue structure [5]. MFS demonstrates clinical variability with predominant involvement of skeletal, cardiovascular, and ocular systems. A variety of skeletal manifestations may be seen due to bone overgrowth and laxity of joints [6]. The cardiovascular complications include aortic root dilatation, aortic dissection, mitral valve prolapse, and enlarged proximal pulmonary artery. These are the major contributors in morbidity and mortality in patients with MFS. Myopia is the most common ocular feature. The ocular hallmark is ectopia lentis that is usually bilateral. Pulmonary complications include bullae and spontaneous pneumothorax; striae atrophicae and incisional hernias are the major skin problems [4].

The gastrointestinal involvement in MFS is rare. There are a few case reports on bowel obstruction due to malrotation of gut, gastroesophageal reflux disease (GERD), small hiatus hernia, gastric volvulus, congenital band, diverticulitis, and Zenker’s diverticula in the adult patient population [7-8]. There is only one previous report of SV with MFS described by Junpaparp et al. [1]. However, no case describing SV in a young patient with concomitant MFS has been reported. Despite the fact that patients with MFS are known to have long sigmoid and cecal mesenteries with possible structural intestinal defects that predispose them to developing a volvulus, such cases are extremely rare. Admittedly, it is difficult to establish this association without a population study, but it would be unusual for a genetic disease that occurs in 1/3000 individuals to be associated with a condition that is not that infrequent as SV.

The new diagnostic criteria for MFS put more weight on the cardiovascular manifestations of the disorder. Aortic root aneurysm and ectopia lentis are now cardinal features. In the absence of any family history, the presence of these two features is sufficient for the unequivocal diagnosis of MFS. In the absence of one of these two cardinal features, the presence of an FBN-1 gene mutation of positive systemic score is required [6]. Diagnosis of MFS was strongly suspected in our patient on the basis of Marfanoid habitus, suggestive family history, echocardiographic evidence of aortic root dilatation and regurgitation, and intraoperative evidence of large redundant sigmoid colon. Family assessment followed by the genetic testing further helped establish the diagnosis.

In MFS patients, the life span is generally reduced, mainly due to the cardiovascular complications. The medical treatment is β-blockers, which decreases the systolic aortic pulse pressure, thereby delaying aortic dilatation [5]. Therefore, MFS patients are also advised not to indulge in strenuous exercise [9].

A multidisciplinary approach, as applied in our case, using clinical, radiologic (CT, plain film radiography), endoscopic, and genetic testing is required for early and accurate diagnosis, which then allows for comprehensive care of such patients, including application of screening measures and interventions to address or prevent serious complications.

## Conclusions

In conclusion, sigmoid volvulus as a presentation of Marfan syndrome is unusual. Clinicians should maintain a high index of suspicion in these patients as prompt diagnosis is life-saving in those with underlying or subsequent cardiovascular complications. Further population-based clinical studies are warranted to broaden the scope of our knowledge on this association and to frame guidelines to standardize the care of such patients.
